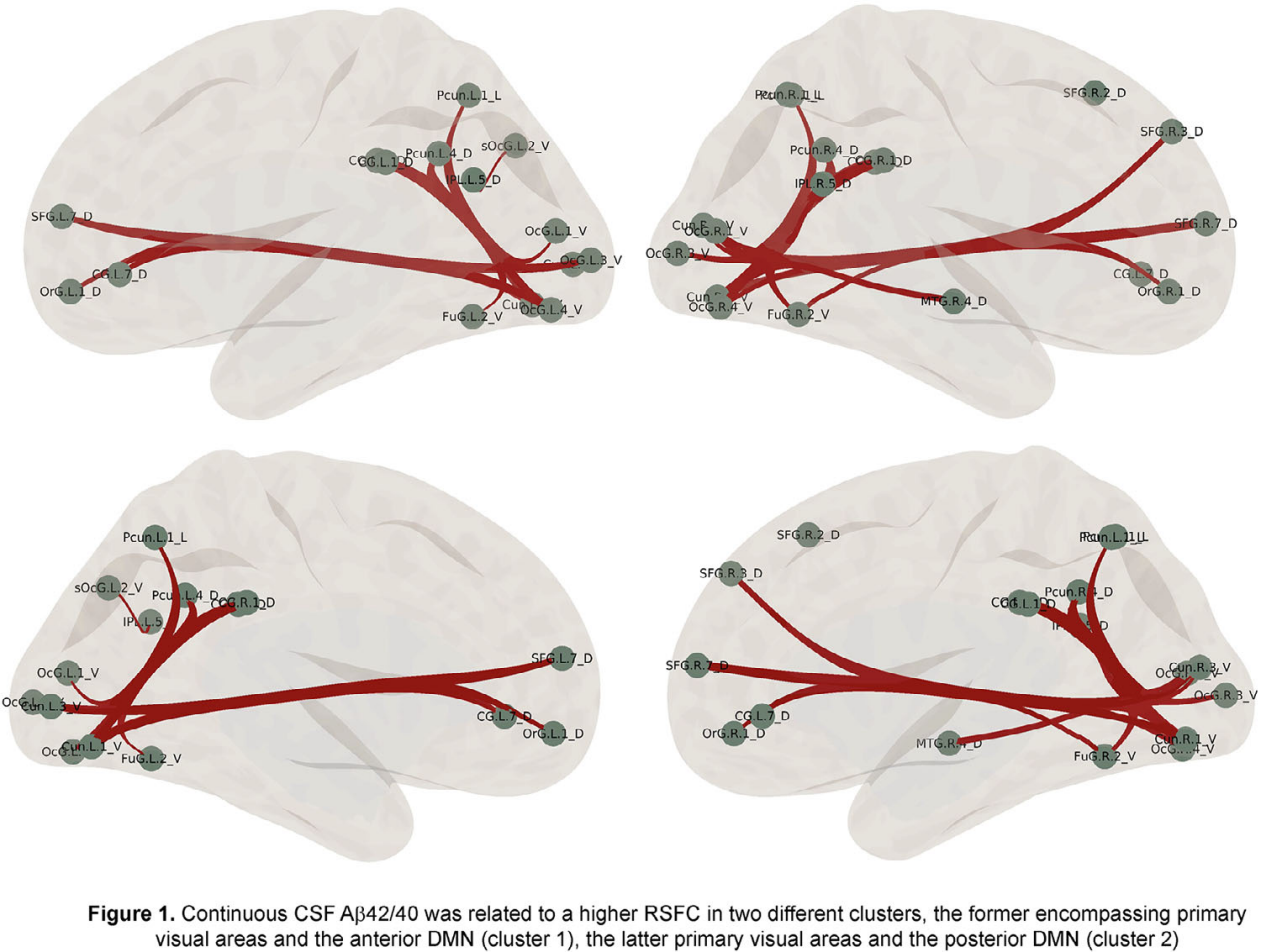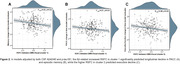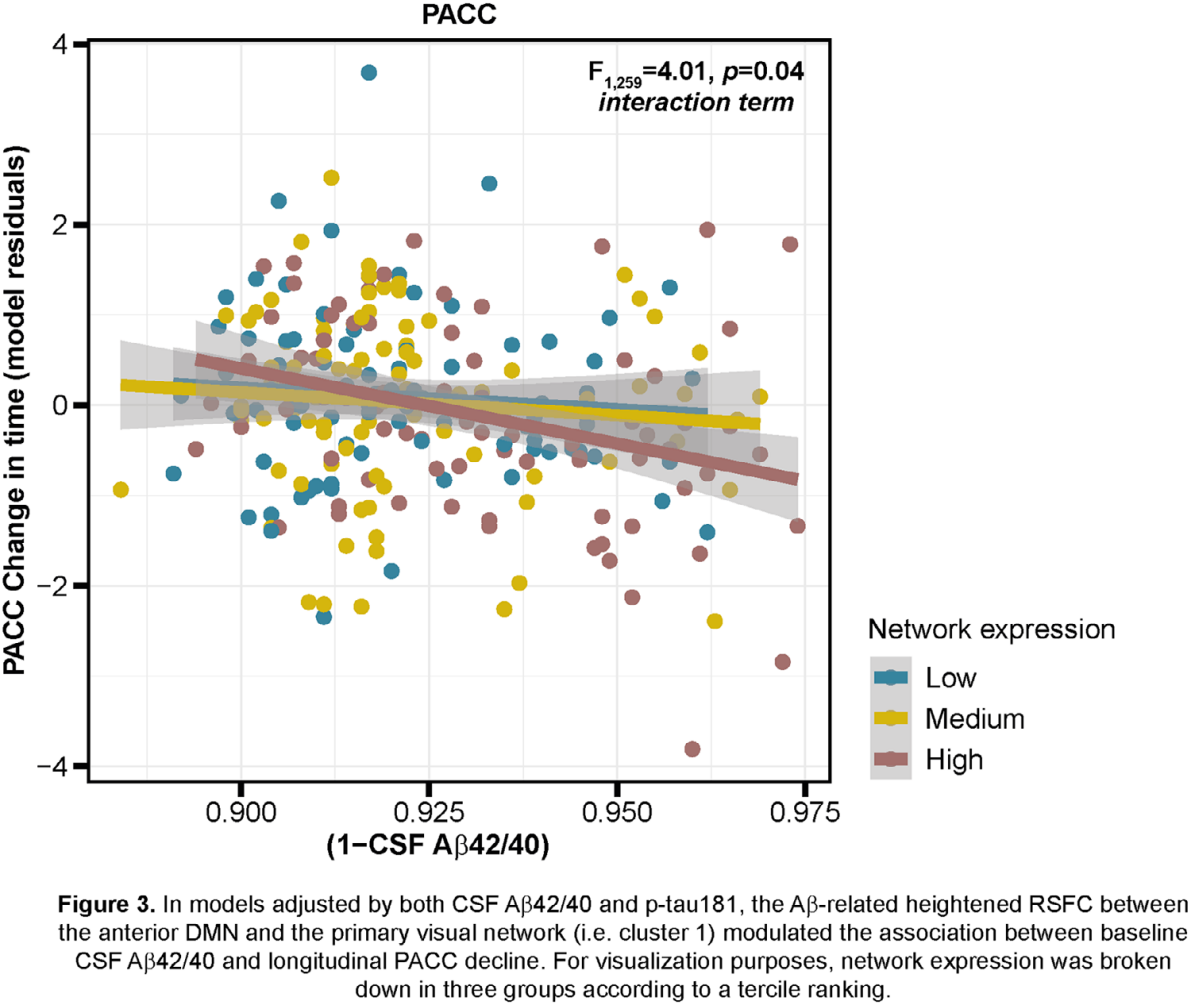# Aberrant resting‐state functional connectivity of the default‐mode network relates to cognitive decline in the earliest Alzheimer's continuum

**DOI:** 10.1002/alz.087302

**Published:** 2025-01-09

**Authors:** Aldana Lizarraga, Michalis Kassinopoulos, José María González‐de‐Echávarri, Jordi Huguet, Gonzalo Sánchez‐Benavides, Anna Brugulat‐Serrat, Marc Suarez‐Calvet, Marta Milà‐Alomà, Kaj Blennow, Henrik Zetterberg, Gwendlyn Kollmorgen, Clara Quijano‐Rubio, Jose Luis Molinuevo, Juan Domingo Gispert, Raffaele Cacciaglia, ALFA study

**Affiliations:** ^1^ Barcelonaβeta Brain Research Center (BBRC), Pasqual Maragall Foundation, Barcelona Spain; ^2^ Hospital del Mar Medical Research Institute (IMIM), Barcelona Spain; ^3^ Centro de Investigación Biomédica en Red de Fragilidad y Envejecimiento Saludable (CIBERFES), Madrid Spain; ^4^ Barcelonaβeta Brain Research Center (BBRC), Barcelona Spain; ^5^ Hospital del Mar Research Institute (IMIM), Barcelona Spain; ^6^ Servei de Neurologia, Hospital del Mar, Barcelona Spain; ^7^ Department of Veterans Affairs Medical Center, Northern California Institute for Research and Education (NCIRE), San Francisco, CA USA; ^8^ Clinical Neurochemistry Laboratory Sahlgrenska University Hospital, Mölndal Sweden; ^9^ Department of Psychiatry and Neurochemistry, Institute of Neuroscience and Physiology, University of Gothenburg, Mölndal Sweden; ^10^ Institute of Neuroscience and Physiology, University of Gothenburg, Mölndal Sweden; ^11^ Department of Psychiatry and Neurochemistry, Institute of Neuroscience and Physiology, The Sahlgrenska Academy at the University of Gothenburg, Mölndal Sweden; ^12^ Hong Kong Center for Neurodegenerative Diseases, Hong Kong China; ^13^ Department of Neurodegenerative Disease, UCL Queen Square Institute of Neurology, University College London, London, ‐ UK; ^14^ Wisconsin Alzheimer's Disease Research Center, School of Medicine and Public Health, University of Wisconsin‐Madison, Madison, WI USA; ^15^ UK Dementia Research Institute at UCL, London UK; ^16^ Roche Diagnostics GmbH, Penzberg Germany; ^17^ Roche Diagnostics International Ltd., Rotkreuz Switzerland; ^18^ Lundbeck A/S, Copenhagen Denmark; ^19^ Hospital del Mar Research Institute, Barcelona, Barcelona Spain; ^20^ Universitat Pompeu Fabra, Barcelona Spain; ^21^ Centro de Investigación Biomédica en Red de Bioingeniería, Biomateriales y Nanomedicina (CIBER‐BBN), Madrid Spain

## Abstract

**Background:**

Amyloid‐β (Aβ) pathology affects resting state functional connectivity (RSFC), even in cognitively unimpaired (CU) individuals. However, the impact of such an aberrant RSFC on cognitive decline is yet to be determined. Moreover, most prior research focused on fibrillary Aβ deposition to predict RSFC, while early Aβ dysmetabolism as reflected by cerebrospinal fluid (CSF) concentrations has received less attention. We assessed RSFC as a function of both CSF Aβ and p‐tau in CU individuals, and further analyzed the impact of biomarker‐dependent RSFC on the longitudinal cognitive performance.

**Method:**

Analyses were conducted in 328 CU individuals from the ALFA cohort (mean age=60.8, SD=4.74) with available CSF Aβ, p‐tau, resting‐state fMRI and longitudinal cognitive assessment (average follow‐up time=3.35 years, SD=0.53). CSF Aβ42 and Aβ40 were assessed with the exploratory NeuroToolKit, while p‐tau181 was measured with the Elecsys® Phospho‐Tau (181P) CSF immunoassay (both Roche Diagnostics International Ltd). RSFC was computed amongst 246 brain regions of the Brainnetome atlas using the CONN toolbox, selecting a cluster threshold of p<0.005. The effects of CSF biomarkers on RSFC were adjusted by age, sex, years of education and APOE‐ε4 status.

**Result:**

Of the entire sample, 38.4% had positive CSF Aβ42/40 markers. Low CSF Aβ42/40 ratios were associated to a higher RSFC between visual areas and anterior as well as posterior subdivisions of the default‐mode network (DMN) (Figure 1). These results survived a family‐wise error rate p‐value<0.005. High levels of CSF p‐tau were related to a higher RSFC between inferior temporal areas and the anterior DMN, as well as a reduced RSFC between visual and the somatomotor network. The Aβ‐related higher RSFC significantly predicted longitudinal cognitive decline in PACC, episodic memory (EM) and executive control (EC), in models adjusted by CSF biomarkers (Figure 2), and further modulated the association between CSF Aβ42/40 and PACC longitudinal decline (Figure 3)

**Conclusion:**

In CU individuals, CSF Aβ and p‐tau affect RSFC in networks relevant for cognitive performance. Low CSF Aβ42/40 was related to hyperconnectivity between the DMN and the visual system. Lack of DMN segregation as a function of CSF Aβ42/40 may represent a driving mechanism of cognitive decline in the earliest Alzheimer’s continuum.